# Supplementary material for: Genome-Wide Analysis of Dental Caries Variability Reveals Genotype-by-Environment Interactions
Source: Genes (Basel). 2023 Mar 17;14(3):736. doi: 10.3390/genes14030736 (PMC10048401; doi:10.3390/genes14030736)
Supplement: Supplementary file 1 [file genes-14-00736-s001.zip › Figure S1.pdf]

vQTL of dfs in the priamry dentition (IFS)

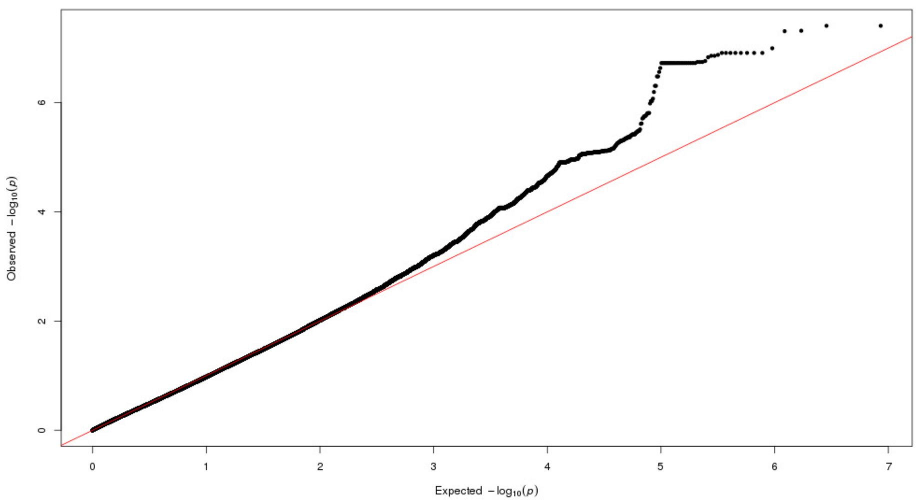

vQTL of dfs in the priamry dentition (COHRA1)

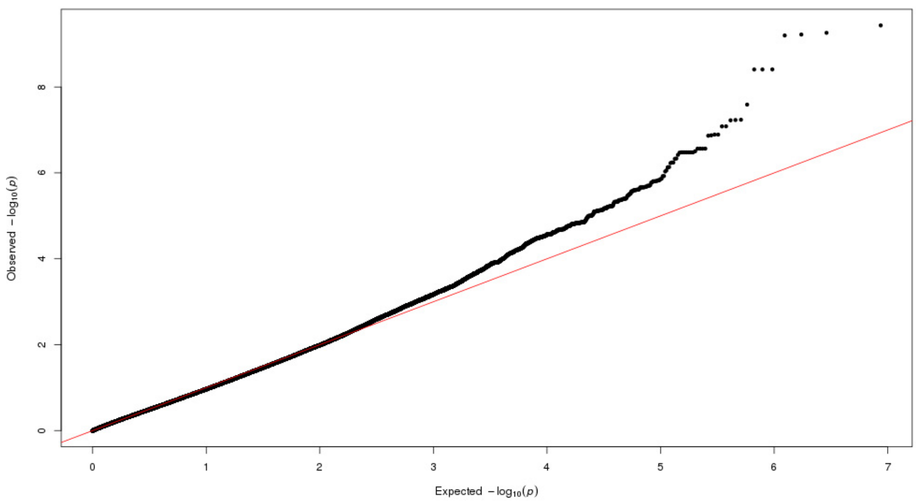

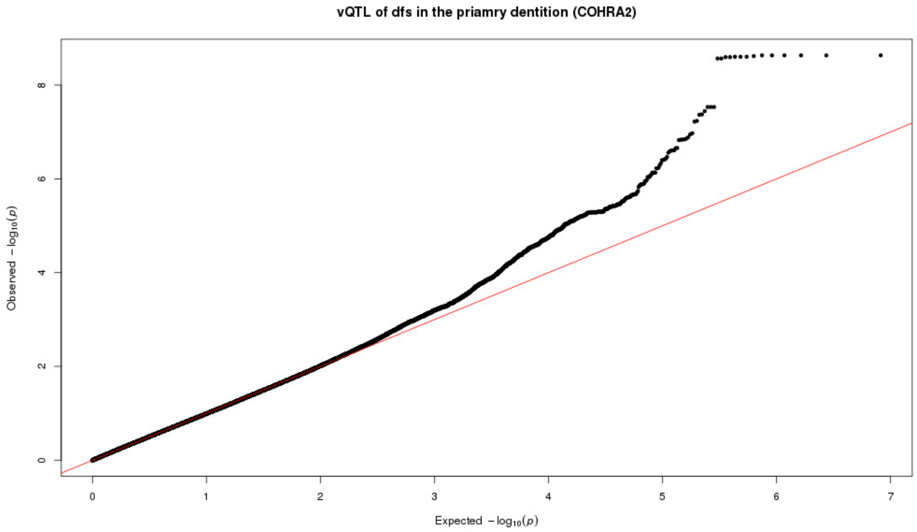

Figure S1 Quantile-quantile plot for genome-wide vQTL analysis of dfs in IFS, COHRA1, and COHRA2

The genomic inflation factors were 1.00, 1.02, and 1.03 in IFS, COHRA1, and COHRA2, repectively.
