# Supplementary material for: Genome-Wide Analysis of Dental Caries Variability Reveals Genotype-by-Environment Interactions
Source: Genes (Basel). 2023 Mar 17;14(3):736. doi: 10.3390/genes14030736 (PMC10048401; doi:10.3390/genes14030736)
Supplement: Supplementary file 1 [file genes-14-00736-s001.zip › Table S1.pdf]

Table S1. P values of interactions between SNPs and factors associated with dfs in IFS

No.	SNP	Sex	Mother edu.	Father edu.	Income	Fluoride level	Brushing	Water source	Birth weight	Gest. weeks	Milk intake	100% juice intake	SSB intake	Fluoride intake	Powder drink intake
1	rs59190052	0.443	0.987	0.113	0.580	0.153	0.355	0.421	0.998	0.370	0.068	0.015	0.040	0.784	0.294
2	rs9830884	0.646	0.360	0.250	**4.24E-05**	0.133	0.277	0.767	0.794	0.709	0.311	0.039	0.148	0.282	0.677
3	rs77322490	0.746	0.051	0.114	0.645	0.936	0.021	0.206	0.708	0.379	0.962	0.497	0.037	0.586	0.854
4	rs6844159	0.851	0.014	0.057	0.051	0.370	0.034	0.050	0.706	0.603	0.275	0.255	0.403	0.401	0.041
5	rs3947271	0.349	0.028	0.028	0.032	0.798	0.052	0.260	0.022	0.448	0.403	0.656	0.475	0.409	0.002
6	rs1089941	0.896	0.058	0.381	0.206	0.654	0.865	0.991	0.580	0.887	0.050	0.179	0.332	0.010	0.199
7	rs1491071	0.326	0.001	**4.25E-06**	0.202	0.991	0.719	0.253	0.416	0.954	0.136	0.813	0.060	0.138	0.195
8	rs2018981	0.092	0.291	0.655	0.605	0.106	0.456	0.143	0.865	0.537	0.081	0.186	0.412	0.160	0.011
9	rs11587481	0.595	0.474	0.495	0.467	0.513	0.493	0.012	0.838	0.402	0.001	0.100	0.638	0.354	0.672
10	rs11199332	0.355	0.081	0.634	0.226	0.202	0.150	0.077	0.874	0.907	0.065	0.605	0.308	0.075	0.773
11	rs11241707	0.263	0.111	0.182	0.022	0.380	0.006	0.366	0.965	0.515	0.377	0.175	0.024	0.100	0.025
12	rs12429729	0.046	0.919	0.913	0.963	0.766	0.070	0.294	0.558	0.525	0.765	0.183	0.741	0.832	0.469
13	rs7463853	0.651	0.074	0.921	0.385	0.292	0.517	0.981	0.207	0.491	0.644	0.816	0.515	0.353	0.330
14	rs690435	0.960	0.134	0.265	0.121	0.989	0.117	0.400	0.083	0.507	0.699	0.486	0.472	0.818	0.036
15	rs12994450	0.108	0.868	0.675	0.453	0.043	0.154	0.122	0.730	0.161	0.519	0.469	0.782	0.936	0.623
16	rs11654217	0.800	0.517	0.790	0.739	0.918	0.750	0.423	0.929	0.147	0.485	0.522	0.873	0.295	0.372
17	rs264532	0.561	0.297	0.589	0.383	0.710	0.326	0.786	0.241	0.552	0.876	0.292	0.600	0.135	0.544
18	rs12797571	0.661	0.619	0.986	0.358	0.145	0.236	0.725	0.443	0.715	0.696	0.445	0.786	0.609	0.273
19	rs11970843	0.859	0.234	0.056	0.179	0.421	0.877	0.346	0.541	0.566	0.861	0.296	0.092	0.627	0.002
20	rs4663531	0.107	0.728	0.054	0.379	0.063	0.372	0.275	0.718	0.257	0.056	0.891	0.640	0.855	0.043
21	rs2090166	0.109	0.270	0.181	0.975	0.303	0.476	0.394	0.695	0.536	0.848	0.731	0.092	0.332	0.034
22	rs3786738	0.629	0.624	0.036	0.137	0.042	0.696	0.940	0.947	0.086	0.337	0.834	0.340	0.900	0.892
23	rs11817228	0.608	0.926	0.139	0.095	0.559	0.652	0.512	0.852	0.814	0.643	0.440	0.353	0.997	0.041
24	rs512158	0.248	0.931	0.961	0.386	0.146	0.473	0.435	0.837	0.660	0.661	0.133	0.872	0.301	0.027
25	rs622516	0.252	0.369	0.601	0.533	0.612	0.845	0.249	0.728	0.943	0.347	0.304	0.342	0.531	0.032
26	rs71508615	0.004	0.984	0.549	0.966	0.788	0.774	0.961	0.039	0.249	0.011	0.904	0.977	0.680	0.594
27	rs9982623	0.083	0.610	0.242	0.414	0.690	0.860	0.204	0.512	0.886	0.902	0.815	0.310	0.847	0.893
28	rs2869342	0.454	0.387	0.384	0.329	0.459	0.306	0.528	0.638	0.186	0.349	0.625	0.542	0.227	0.684
29	rs17536922	0.674	0.260	0.124	0.294	0.083	0.470	0.101	0.520	0.686	0.329	0.112	0.128	0.814	0.554
30	rs10651815	0.177	0.593	0.829	0.514	0.806	0.399	0.567	0.654	0.415	0.003	0.752	0.954	0.687	0.333
31	rs1958016	0.478	0.482	0.517	0.779	0.822	0.739	0.692	0.281	0.037	0.298	0.557	0.343	0.458	0.160

32	rs73723358	0.542	0.735	0.343	0.813	0.156	0.301	0.263	0.915	0.582	0.978	0.301	0.289	0.743	0.501
33	rs7972868	0.771	0.562	0.972	0.287	0.406	0.983	0.980	0.634	0.540	0.890	0.669	0.064	0.977	0.039
34	rs73157913	0.792	0.221	0.611	0.143	0.491	0.898	0.488	0.926	0.743	0.405	0.343	0.217	0.566	0.602
35	rs11923408	0.507	0.657	0.897	0.283	0.220	0.591	0.623	0.730	0.951	0.915	0.685	0.863	0.054	0.225
36	rs9685188	0.672	0.853	0.552	0.408	0.469	0.089	0.010	0.722	0.233	0.736	0.535	0.725	0.498	0.413
37	rs3862191	0.109	0.270	0.181	0.975	0.303	0.476	0.394	0.695	0.536	0.848	0.731	0.092	0.332	0.034
38	rs11592458	0.551	0.792	0.218	0.060	0.662	0.151	0.153	0.633	0.487	0.679	0.532	0.005	0.096	0.842
39	rs1497945	0.183	0.390	0.529	0.203	0.420	0.612	0.541	0.412	0.386	0.138	0.083	0.507	0.190	0.007
40	rs1978471	0.045	0.002	0.002	0.076	0.628	**3.15E-05**	0.325	0.374	0.584	0.057	0.815	0.311	0.502	0.001

Note: Bolded numbers are the significant GEI. Income: household income; Mother edu.: mother's educational attainment; Father edu.: father's educational attainment; Brushing: Toothbrushing frequency; Gest.weeks: Gestational weeks; Powder drink intake: Powdered beverage intake.
