# Supplementary material for: Genome-Wide Analysis of Dental Caries Variability Reveals Genotype-by-Environment Interactions
Source: Genes (Basel). 2023 Mar 17;14(3):736. doi: 10.3390/genes14030736 (PMC10048401; doi:10.3390/genes14030736)
Supplement: Supplementary file 1 [file genes-14-00736-s001.zip › Table S2.pdf]

Table S2. P values of interactions between SNPs and factors associated with dfs in COHRA1

No	SNP	Sex	Site	Mother edu.	Father edu.	Mother income	Father income	Fluoride. level	Brushing	Water Source	Fluoride supplement
1	rs59190052	0.616	0.956	0.182	0.063	0.884	0.956	0.216	0.563	0.851	0.077
2	rs9830884	0.757	0.104	0.560	0.264	0.305	0.354	0.566	0.515	0.126	0.757
3	rs77322490	0.258	0.874	0.092	0.305	0.800	0.776	0.013	0.348	0.731	0.751
4	rs6844159	0.651	0.609	0.542	0.574	0.163	0.876	0.101	0.225	0.697	0.595
5	rs3947271	0.678	0.289	0.108	0.344	0.636	0.886	0.698	0.472	0.565	0.667
6	rs1089941	0.674	0.074	0.410	0.235	0.223	0.773	0.951	0.109	0.412	0.694
7	rs1491071	0.619	0.925	0.641	0.432	0.599	0.483	0.828	0.655	0.537	0.053
8	rs2018981	0.253	0.807	0.781	0.344	0.468	0.104	0.688	0.467	0.711	0.315
9	rs11587481	0.386	0.127	0.222	0.707	0.771	0.866	0.820	0.911	0.568	0.011
10	rs11199332	0.081	0.698	0.476	0.933	0.535	0.936	0.431	0.025	0.649	0.858*
11	rs11241707	0.648	0.724	0.222	0.698	0.593	0.651	0.970	0.234	0.854	0.474
12	rs12429729	0.353	0.004	0.023	0.003	0.008	0.003	0.954	0.030	0.001	0.887
13	rs7463853	0.418	0.021	0.167	3.1E-04	0.033	**3.50E-05**	0.688	0.047	0.135	0.06
14	rs690435	0.936	0.165	0.841	0.062	0.209	0.192	0.434	0.060	0.005	0.156
15	rs12994450	0.312	0.869	0.711	0.177	0.137	0.001	0.093	0.057	0.124	0.091
16	rs11654217	0.634	0.023	0.004	0.083	0.055	0.067	0.897	0.320	0.128	0.217
17	rs264532	0.861	0.384	0.616	0.286	0.901	0.090	0.003	0.005	4.5E-04	0.113*
18	rs12797571	0.296	0.263	0.084	0.016	0.150	0.003	0.943	0.258	0.050	0.737
19	rs11970843	0.514	0.418	0.004	0.482	0.537	0.129	0.925	0.711	0.721	0.042
20	rs4663531	0.021	0.294	0.457	0.262	0.295	0.874	0.102	0.462	0.012	0.802
21	rs2090166	0.330	0.405	0.108	0.811	0.402	0.229	0.054	0.354	0.403	0.716
22	rs3786738	0.877	0.817	0.836	0.806	0.064	0.191	0.744	0.311	0.207	0.824
23	rs11817228	0.528	0.339	0.913	0.952	0.170	0.646	0.704	0.743	0.674	0.589
24	rs512158	0.828	0.921	0.697	0.922	0.597	0.603	0.647	0.965	0.760	0.259
25	rs622516	0.649	0.483	0.670	0.977	0.594	0.424	0.107	0.272	0.236	0.093
26	rs71508615	0.378	0.861	0.866	0.335	0.556	0.555	0.651	0.084	0.295	0.77
27	rs9982623	0.406	0.155	0.494	0.320	0.220	0.677	0.622	0.848	0.307	0.125
28	rs2869342	0.755	0.865	0.517	0.642	0.540	0.928	0.117	0.330	0.293	0.164
29	rs17536922	0.449	0.644	0.788	0.802	0.506	0.243	0.865	0.208	0.346	0.134*
30	rs10651815	0.856	0.681	0.174	0.860	0.821	0.227	0.101	0.181	0.158	0.582
31	rs1958016	0.109	0.924	0.914	0.894	0.704	0.977	0.121	0.909	0.432	0.222
32	rs73723358	0.334	0.343	0.103	0.121	0.768	0.195	0.394	0.951	0.240	0.791*

33	rs7972868	0.714	0.774	0.224	0.468	0.869	0.961	0.666	0.099	0.291	0.348
34	rs73157913	0.472	0.945	0.176	0.687	0.532	0.679	0.157	0.440	0.903	0.946*
35	rs11923408	0.314	0.545	0.505	0.902	0.381	0.570	0.256	0.298	0.545	0.614
36	rs9685188	0.602	0.679	0.444	0.885	0.899	0.791	0.497	0.239	0.328	0.535
37	rs3862191	0.366	0.399	0.103	0.795	0.414	0.219	0.068	0.344	0.413	0.7
38	rs11592458	0.244	0.085	0.685	0.428	0.630	0.396	0.370	0.602	0.306	0.631
39	rs1497945	0.458	0.342	0.590	0.410	0.172	0.161	0.295	0.915	0.267	0.875
40	rs1978471	0.804	0.256	0.236	0.218	0.019	0.078	0.578	0.326	0.021	0.109

Note: Bolded numbers are the significant GEI. Mother edu.: mother's educational attainment; Father edu.: father's educational attainment; Brushing: Toothbrushing frequency.

* There were no participants having two effect alleles of these SNPs and taking fluoride supplement. For these SNPs, we combined genotype groups of two effect alleles and one affect allele, then compared to zero affect allele.
