# Supplementary material for: Genome-Wide Analysis of Dental Caries Variability Reveals Genotype-by-Environment Interactions
Source: Genes (Basel). 2023 Mar 17;14(3):736. doi: 10.3390/genes14030736 (PMC10048401; doi:10.3390/genes14030736)
Supplement: Supplementary file 1 [file genes-14-00736-s001.zip › Table S3.pdf]

Table S3. P values of interactions between SNPs and factors associated with dfs in COHRA2

No	SNP	Sex	Site	Mother edu.	Mother income	Water source	Fluoride level	Brushing	BF.duration	BF.status	SSB1	SSB2
1	rs59190052	0.151	0.327	0.637	0.565	0.211	0.693	0.865	0.884	0.050	0.492	0.438
2	rs9830884	0.104	0.170	0.049	0.281	0.025	0.683	0.853	0.334	0.607	0.997	0.742
3	rs77322490	0.390	0.469	0.862	0.911	0.013	0.684	0.776	0.368	0.858	0.137	0.117
4	rs6844159	0.001	0.275	0.966	0.232	0.395	0.697	0.027	0.285	0.211	0.812	0.815
5	rs3947271	0.042	0.522	0.889	0.907	0.287	0.695	0.444	0.695	0.578	0.091	0.077
6	rs1089941	0.089	0.555	0.061	0.045	0.006*	0.291	0.189	0.793	0.102	0.079	0.338
7	rs1491071	0.978	0.895	0.205	0.144	0.499*	0.685	0.423	0.422	0.172	0.026	0.214
8	rs2018981	0.776	0.729	0.807	0.856	0.713	0.699	0.658	0.832	0.295	0.097	0.157
10	rs11199332	0.627	0.747	0.818	0.287	0.021*	0.692	0.550	0.067	0.942	0.607	0.693
11	rs11241707	0.703	0.928	0.156	0.611	0.097	0.690	0.742	0.262	0.218	0.581	0.631
12	rs12429729	0.499	0.750	0.899	0.807	0.266*	0.694	0.302	0.403	0.541	0.315	0.257
13	rs7463853	0.201	0.263	0.049	0.510	0.107*	0.896	0.337	0.726	0.298	0.729	0.542
14	rs690435	0.102	0.272	0.056	0.650	0.028	0.274	0.004	0.039	0.902	0.043	0.186
15	rs12994450	0.901	0.286	0.599	0.662	0.083	0.689	0.287	0.930	0.330	0.958	0.893
16	rs11654217	0.670	0.239	0.627	0.252	0.761*	0.601	0.335	0.811	0.281	0.661	0.925
17	rs264532	0.350	0.321	0.309	0.734	0.020	0.694	0.359	0.268	0.021	0.128	0.241
18	rs12797571	0.782	0.917	0.063	0.577	0.843	0.400	0.541	0.224	0.146	0.176	0.262
20	rs4663531	0.490	0.749	0.334	0.993	0.191	0.277	0.630	0.689	0.564	0.008	0.062
21	rs2090166	0.418	0.388	0.326	0.916	0.343	0.289	0.569	0.725	0.503	0.050	0.441
22	rs3786738	0.195	0.452	0.111	0.190	0.506	0.689	0.512	0.170	0.004	0.001	0.021
23	rs11817228	0.043	0.735	0.604	0.090	0.850	0.690	0.660	0.040	0.262	0.360	0.345
24	rs512158	0.402	0.185	0.007	0.259	0.010	0.267	0.479	0.628	0.522	0.004	0.002
25	rs622516	0.00037	0.063	0.060	0.013	0.290*	0.683	0.407	0.523	0.058	0.458	0.957
26	rs71508615	**6.78E-06**	0.735	0.00037	**1.25E-04**	0.150	0.678	0.029	0.003	0.041	0.089	0.194
27	rs9982623	0.186	0.077	0.969	0.094	0.173	0.381	0.812	0.289	0.833	0.241	0.180
28	rs2869342	0.751	0.902	0.022	0.600	0.314	0.684	0.801	0.118	2.4E-04	0.817	0.692
29	rs17536922	0.116	0.957	0.390	0.260	0.576	0.676	0.886	0.296	0.511	0.861	0.384
30	rs10651815	0.006	0.371	0.161	0.185	0.193	0.240	0.680	0.686	0.305	0.241	0.292
31	rs1958016	0.648	0.174	0.055	0.060	0.187*	0.682	0.601	0.801	0.608	0.753	0.781
32	rs73723358	0.775	0.003	0.004	0.00014	0.022	0.676	0.461	0.052	0.814	**7.39E-05**	0.047
33	rs7972868	0.025	0.731	0.037	0.004	0.358	0.689	0.835	0.957	0.003	0.070	0.021
34	rs73157913	0.337	0.237	0.223	0.433	0.047*	0.687	0.060	0.787	0.465	0.054	0.019

35	rs11923408	0.354	0.381	0.919	0.925	0.443	0.686	0.657	0.932	0.328	0.098	0.251
36	rs9685188	0.175	0.783	0.001	0.007	0.858	0.686	0.424	0.556	**8.03E-05**	0.00033	0.004
37	rs3862191	0.472	0.394	0.343	0.938	0.343	0.289	0.575	0.801	0.433	0.059	0.510
38	rs11592458	0.025	0.162	0.006	0.123	0.064	0.689	0.684	0.115	0.207	0.614	0.914
39	rs1497945	0.201	0.449	0.027	0.780	0.022	0.405	0.612	0.174	0.302	0.255	0.399
40	rs1978471	0.216	0.513	0.631	0.149	0.751	0.607	0.769	0.153	0.695	0.934	0.832

Note: Bolded numbers are the significant GEI. Mother edu.: mother's educational attainment; Brushing: Toothbrushing frequency; BF.duration: Breastfeeding duration; BF.status: Breastfeeding status; SSB: sugar-sweetened beverages.

* There were no participants having two effect alleles of these SNPs and with water source from well. For these SNPs, we combined genotype groups of two effect alleles and one affect allele, then compared to zero affect allele.
