# Supplementary figures and images for: Genome-Wide Analysis of Dental Caries Variability Reveals Genotype-by-Environment Interactions
Source: Genes (Basel). 2023 Mar 17;14(3):736. doi: 10.3390/genes14030736 (PMC10048401; doi:10.3390/genes14030736)

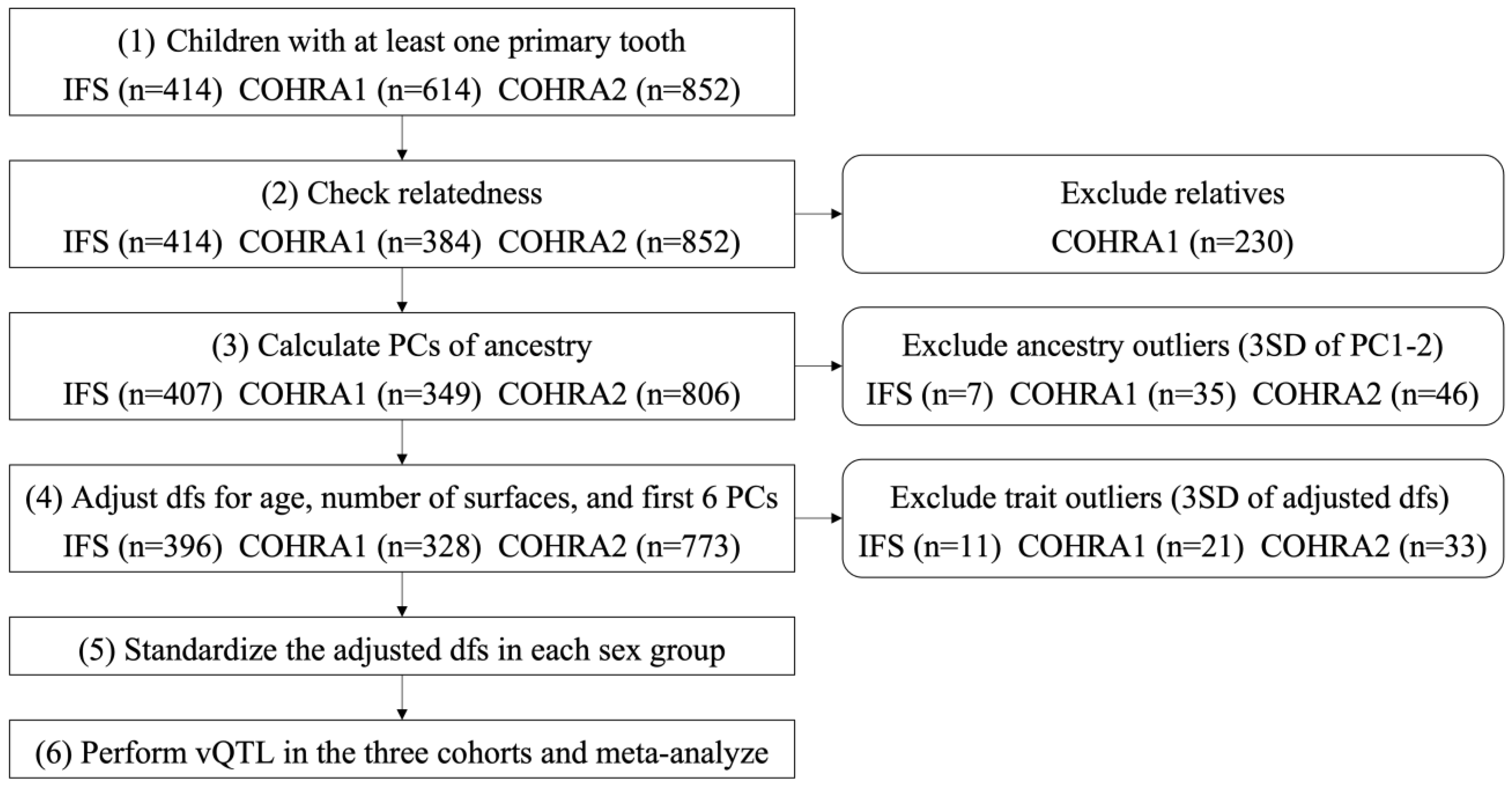

Supplement: Supplementary file 1 [file genes-14-00736-s001.zip › genes-2231568-g001.tif]

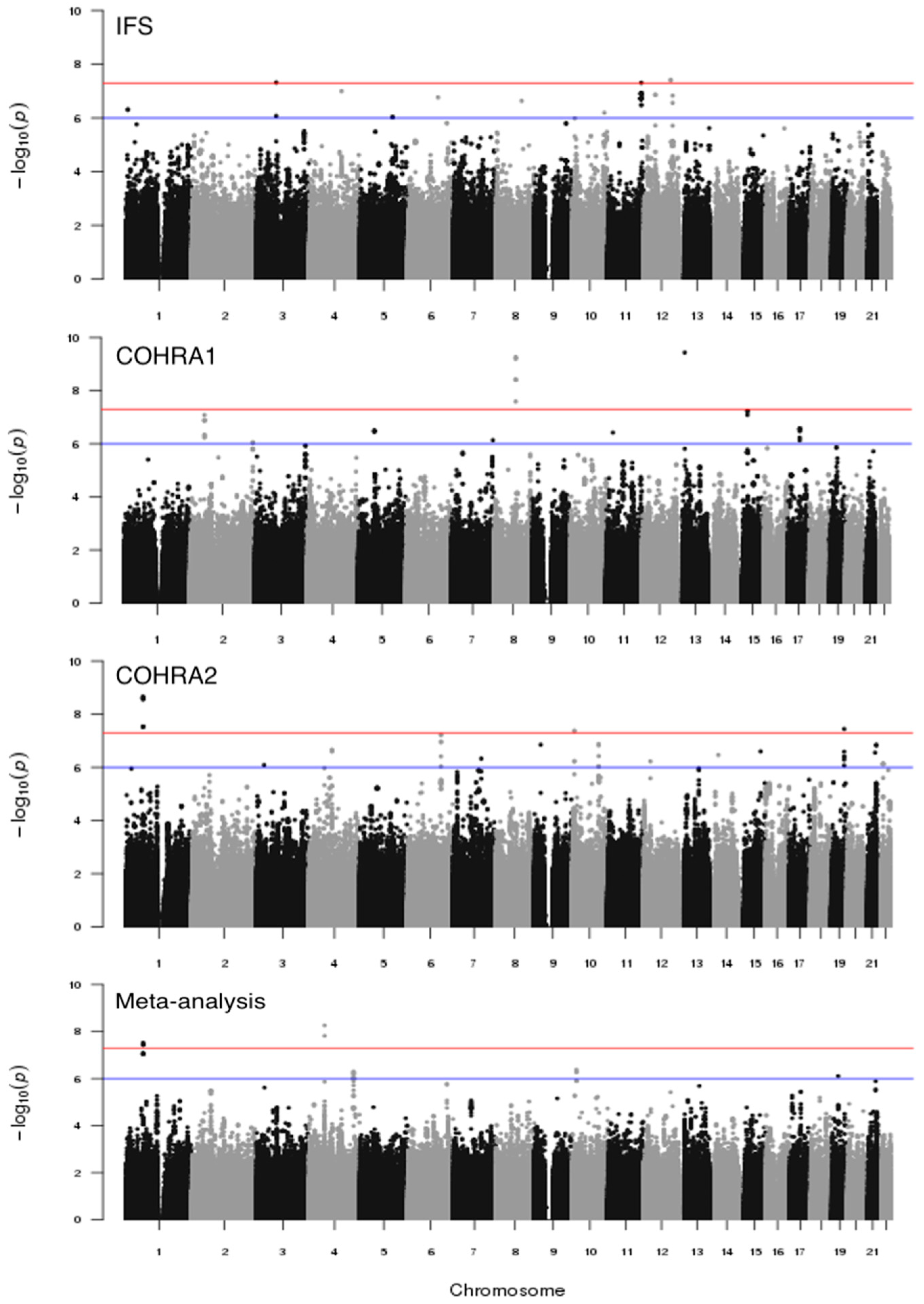

Supplement: Supplementary file 1 [file genes-14-00736-s001.zip › genes-2231568-g002.tif]

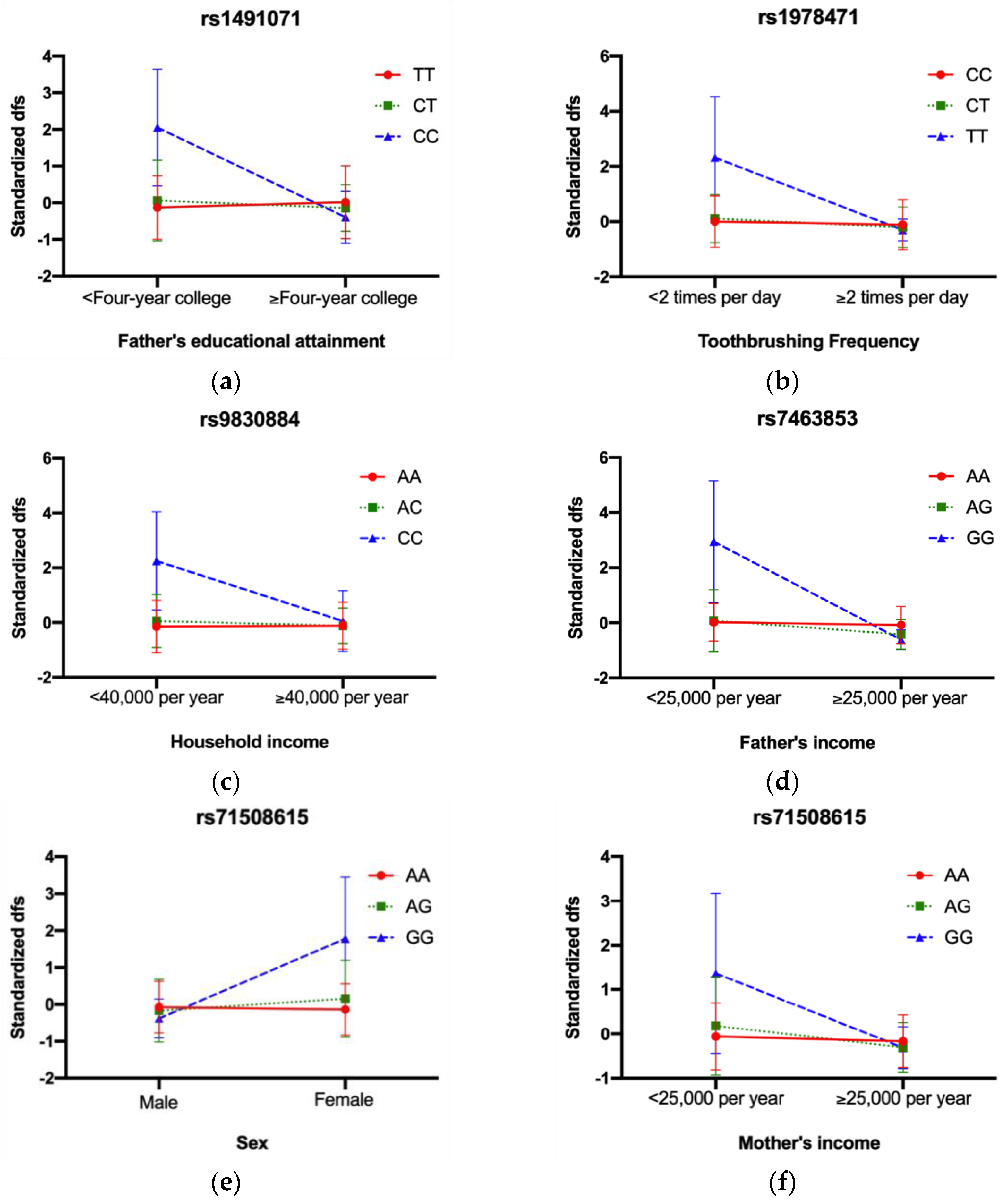

Supplement: Supplementary file 1 [file genes-14-00736-s001.zip › genes-2231568-g003a.tif]

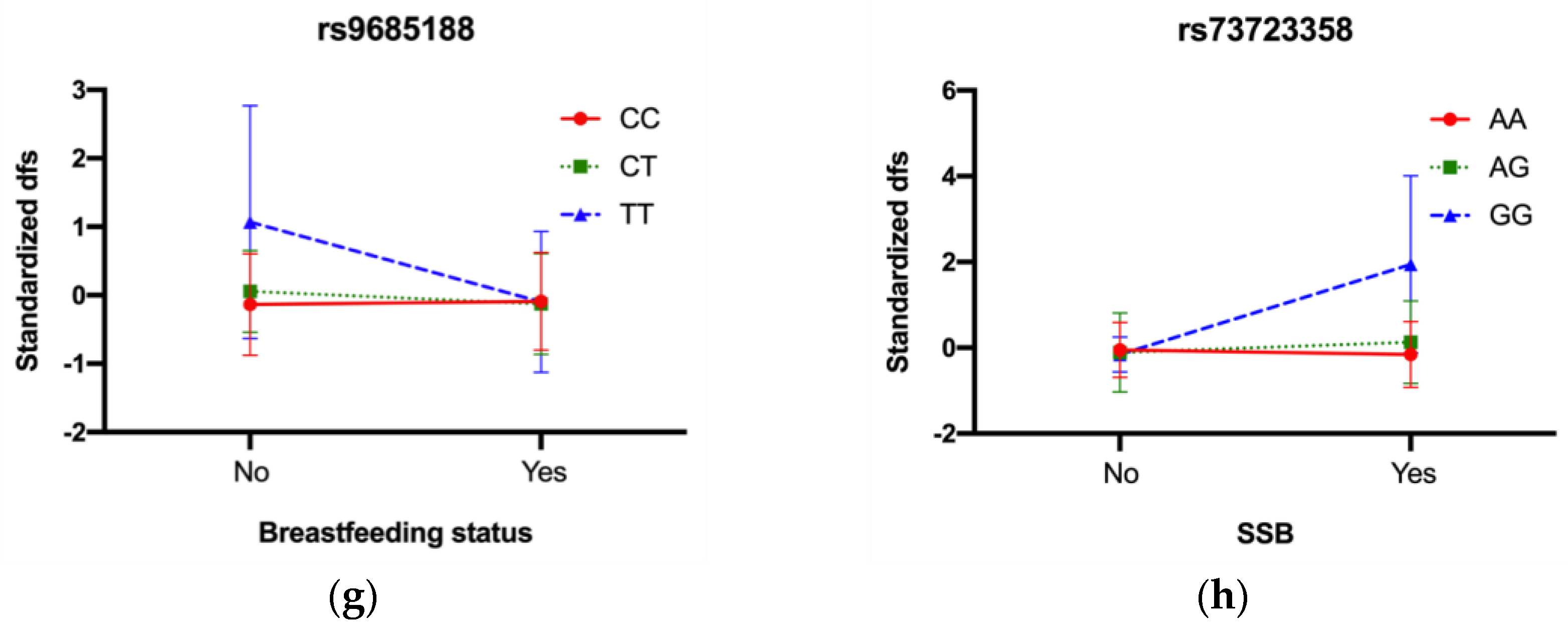

Supplement: Supplementary file 1 [file genes-14-00736-s001.zip › genes-2231568-g003b.tif]
